# Extracellular Matrix-Dependent Generation of Integration- and Xeno-Free iPS Cells Using a Modified mRNA Transfection Method

**DOI:** 10.1155/2016/6853081

**Published:** 2016-01-12

**Authors:** Kang-In Lee, Seo-Young Lee, Dong-Youn Hwang

**Affiliations:** Department of Biomedical Sciences, CHA University, Seongnam, Gyeonggi-do, Republic of Korea

## Abstract

Human induced pluripotent stem cells (iPS cells) hold great promise in the field of regenerative medicine, especially immune-compatible cell therapy. The most important safety-related issues that must be resolved before the clinical use of iPS cells include the generation of “footprint-free” and “xeno-free” iPS cells. In this study, we sought to examine whether an extracellular matrix- (ECM-) based xeno-free culture system that we recently established could be used together with a microRNA-enhanced mRNA reprogramming method for the generation of clinically safe iPS cells. The notable features of this method are the use of a xeno-free/feeder-free culture system for the generation and expansion of iPS cells rather than the conventional labor-intensive culture systems using human feeder cells or human feeder-conditioned medium and the enhancement of mRNA-mediated reprogramming via the delivery of microRNAs. Strikingly, we observed the early appearance of iPS cell colonies (~11 days), substantial reprogramming efficiency (~0.2–0.3%), and a high percentage of ESC-like colonies among the total colonies (~87.5%), indicating enhanced kinetics and reprogramming efficiency. Therefore, the combined method established in this study provides a valuable platform for the generation and expansion of clinically safe (i.e., integration- and xeno-free) iPS cells, facilitating immune-matched cell therapy in the near future.

## 1. Introduction

The discovery of induced pluripotent stem cells (iPS cells) has opened a new avenue for patient-specific and immune-compatible cell replacement therapy [1].

The initial approaches used to introduce reprogrammed genes to human fibroblasts relied on retroviral or lentiviral vectors, which caused undesired random insertion of transgenes into chromosomes [2, 3]. The chromosomal integration of transgenes by these viral vectors potentially causes tumor formation depending on the insertion sites, as clearly demonstrated in previous gene therapy trials for X-linked severe combined immunodeficiency [4–6]. Furthermore, the integrated transgenes may be continuously expressed after reprogramming due to incomplete silencing or, in some cases, may elicit full expression resulting from reactivation.

Therefore, methodologies for generating iPS cells without chromosomal integration of exogenous reprogrammed genes have been evolving rapidly. These methods include episomal plasmid transfection [7–9], Sendai virus-mediated gene delivery [10], and mRNA transfection [11].

Among these three integration-free methods, the mRNA transfection method displays several unique advantages. For example, in contrast to episomal plasmid transfection, mRNA transfection completely avoids the possibility of chromosomal integration. In addition, unlike both episomal plasmid transfection and Sendai viral infection, mRNA transfection does not require prolonged passaging to remove lingering exogenous gene expression due to the short half-life of the introduced mRNAs. However, the requirement of 17 consecutive daily transfections of mRNAs [11, 12] is highly laborious, which potentially limits the utility of this method for producing Good Manufacturing Practice- (GMP-) grade iPS cells for cell therapy. Therefore, it is desirable to establish a more efficient and convenient method to generate iPS cells using mRNAs.

Another important issue to consider regarding the clinical application of iPS cells is the generation and expansion of these cells under strictly xeno-free conditions. Xeno-free culture prevents xenopathogen transmission and immune complications caused by non-human antigens [13, 14]. To perform mRNA-mediated reprogramming, the initial and subsequent studies used human feeder cells, and human neonatal fibroblast- (NuFF-) conditioned medium [11, 12, 15, 16]. Although these methods used xeno-free conditions during reprogramming, the preparation of human feeder cells or human feeder-conditioned medium is cumbersome and labor-intensive. Therefore, there has been great demand for the establishment of a simpler and more convenient mRNA-mediated reprogramming protocol for cell replacement therapy.

In this study, we sought to establish such a method by combining our previously established extracellular matrix- (ECM-) based xeno-free/feeder-free human pluripotent stem cell (hPSC) culture system [17] with an improved mRNA-mediated reprogramming protocol. Because clinically safe iPS cells are required for cell replacement therapy, this study provides a useful platform that facilitates future cell therapeutic approaches using iPS cells.

## 2. Materials and Methods

### 2.1. Cell Culture

The study was approved by the Ethical Committee of the CHA University Bundang CHA Hospital, Republic of Korea (application number: KNC12005). Human adult dermal fibroblasts (ScienCell Research Laboratories, Carlsbad, CA, USA) were cultured in DMEM (WelGENE, Daegu, Korea) supplemented with 10% fetal bovine serum (FBS), 2 mM L-glutamine (Invitrogen) and 1x penicillin/streptomycin (P/S) (all from Invitrogen, Carlsbad, CA, USA).

Human iPS cells were cultured on vitronectin XF (Primorigen Biosciences, Madison, USA) coated culture dishes using our recently established xeno-free/feeder-free hPSC culture medium with minor modifications [17]. Briefly, the medium consisted of DMEM/F12, 15% KnockOut SR XenoFree CTS, 1x nonessential amino acids (NEAA), 1x GlutaMAX, 0.1 mM *β*-mercaptoethanol, 1x P/S (all from Invitrogen), 10 ng/mL basic fibroblast growth factor (bFGF) (CHA Biotech Co., Daejeon, Korea), 10 nM trichostatin A (TSA) (Sigma-Aldrich, St. Louis, MO, USA), 5 uM Gö6983 (Tocris, Ellisville, MO, USA), and 1 mM dorsomorphin dihydrochloride (Tocris).

### 2.2. The Generation of Integration- and Xeno-Free iPS Cells

The modified mRNA-based reprogramming reagents used in this study were a kind gift from Stemgent, Inc. (Cambridge, MA, USA). This system consisted of a microRNA cocktail solution (20 uM) containing microRNAs that perform reprogramming functions, including mir302a-d and mir367, and an mRNA cocktail containing mRNAs for Oct4, Sox2, Klf4, c-Myc, and Lin28 at a molar stoichiometry of 3 : 1 : 1 : 1 : 1, respectively. Each mRNA was included at a concentration of 100 ng/*μ*L. The generation of iPS cells using the reagents was performed according to the protocol provided by Stemgent, Inc., with minor modifications [11].

To generate iPS cells, first, human adult dermal fibroblasts were seeded at a density of 5 × 10^4^ cells per well in a 6-well dish precoated with vitronectin XF (10 *µ*g/mL). The next day, the culture medium was replaced with our xeno-free/feeder-free hPSC culture medium 2 hours before transfection. Recombinant B18R protein (working concentration, 200 ng/*µ*L) (eBioscience, Inc., San Diego, CA, USA), a type 1 interferon inhibitor, was added at this time point to promote cell viability after RNA transfection. A Stemfect RNA Transfection Kit (Stemgent, Inc.) was used for RNA transfection: 1 *µ*g of the mRNA cocktail and/or 3.5 *µ*L of a microRNA cocktail (20 *µ*M) was mixed with Stemfect Transfection Buffer in one tube (total volume of 50 *µ*L), and 4 *µ*L of the Stemfect RNA Transfection Reagent was added to the Stemfect Transfection Buffer in a second tube (total volume of 50 *µ*L). The mixture in the second tube was added to the first tube, followed by gentle pipetting of the total 100 *µ*L volume of the combined solution. After incubating the RNA-liposome complex for 15 minutes at room temperature, the mixture was added to the medium in a drop-wise manner with gentle shaking of the dish to ensure uniform distribution of the RNA-liposome complex in the wells. After incubation of the cells for 4 hours, the medium was replaced with 2 mL of fresh xeno-free/feeder-free hPSC culture medium. mRNA transfection was conducted for 11 consecutive days beginning on day 2, and microRNA transfection was performed on days 1 and 5 after cell seeding (twice only). The appearance of iPS cell colonies was monitored every day, and an individual colony was selected at approximately days 20–22 via mechanical methods for further clonal expansion.

### 2.3. Quantitative Reverse Transcriptase-Polymerase Chain Reaction (qRT-PCR)

Total RNA was isolated using a NucleoSpin RNA II Kit (Macherey-NagelGmbH & Co. KG, Duren, Germany) according to the manufacturer's instructions. Complementary DNA (cDNA) was synthesized from 1 *µ*g of total RNA using a ReverTra Ace qPCR RT Kit (Toyobo, Osaka, Japan), and qRT-PCR analysis was performed using Power SYBR Green PCR Master Mix (Applied Biosystems, Foster City, CA, USA) and a StepOnePlus Real-Time PCR System (Applied Biosystems) under the following conditions: 40 cycles of DNA denaturation at 95°C for 5 seconds, DNA annealing with each primer pair at 55–63°C for 30 seconds, and polymerization at 72°C for 30 seconds. The human *β*-actin gene was used as a normalization control. The primers used in the qRT-PCR experiments are listed in Supplementary Table  1 available online at http://dx.doi.org/10.1155/2016/6853081.

### 2.4. *In Vitro* Differentiation Assay

To test their pluripotency, the iPS cell colonies were mechanically detached and cultured in suspension in Petri dishes (SPL Lifesciences, Pocheon, Korea) in embryoid body (EB) medium (DMEM/F12, 10% KnockOut SR XenoFree CTS, 1x NEAA, 1x P/S, and 0.1 mM *β*-mercaptoethanol; all from Invitrogen). After 5–10 days of 3-dimensional culturing, the EBs were attached to Matrigel (BD Biosciences, Bedford, MA, USA) coated slides and further cultured for 15 days in differentiation medium (DMEM/F12 supplemented with 1% NEAA, 1x P/S, 0.1 mM *β*-mercaptoethanol, and 10% FBS for endoderm and mesoderm or DMEM/F12 supplemented with 1x NEAA, 1% P/S, 0.1 mM *β*-mercaptoethanol, 1x N2 supplement, and 10 ng of bFGF for ectoderm). Several representative markers specific for derivatives of these three germ layers were used for immunostaining after differentiation. The antibodies used in this study are listed in Supplementary Table  2.

### 2.5. Teratoma Formation

Approximately 2 × 10^6^ iPS cells per mouse were injected intramuscularly into the thigh of NOD/SCID mice, and the tumor masses were dissected at 9–12 weeks after injection. The presence of all three germ layer structures in the tumor masses was examined after staining with hematoxylin and eosin.

### 2.6. Karyotype Analysis

G-banding analysis of the iPS cells harvested from a T-25 flask was performed at SamKwang Medical Laboratories (Smlab, Seoul, Korea).

### 2.7. DNA Fingerprinting

To confirm that the iPS cells did indeed originate from the fibroblasts used for iPS cell generation, DNA fingerprints of the genomic DNA from the iPS cells and the fibroblasts were compared at the Korea Gene Information Center (Seoul, Korea).

### 2.8. Bisulfite Sequencing

The genomic DNA from human fibroblasts, H9-hESCs, and iPS cells was extracted using an Exgene Tissue SV kit (GeneAll Biotechnology, Seoul, Korea), and the DNAs were treated with an EpiTech Bisulfite Kit (Qiagen GmbH, Hilden, Germany) for bisulfite conversion according to the manufacturer's instructions. The “CpG-rich” promoter regions of the human Oct4 and Nanog genes were amplified using the specific PCR primers listed in S1 Table. The amplified PCR products were subcloned into the TA cloning vector (RBC Bioscience Corp., New Taipei City, Taiwan) and were subjected to sequencing analysis.

### 2.9. Global Gene Expression Profiling

Total RNA samples were prepared using a NucleoSpin RNA II Kit (Mahcerey-Nagel GmbH & Co. KG, Duren, Germany) according to the manufacturer's protocol. The profiling of global gene expression was performed at Macrogen, Inc. (Seoul, Korea) using 2 *µ*g of total RNA and the HumanHT-12 v4 Expression BeadChip (Illumina, Inc., San Diego, CA, USA). Microarray data was deposited in a public repository such as gene expression omnibus (GEO, series record GSE68035).

## 3. Results

### 3.1. Generation of Xeno- and Footprint-Free iPS Cells via RNA Transfection in an ECM-Based Feeder-Independent Culture System

**Figure 1:**
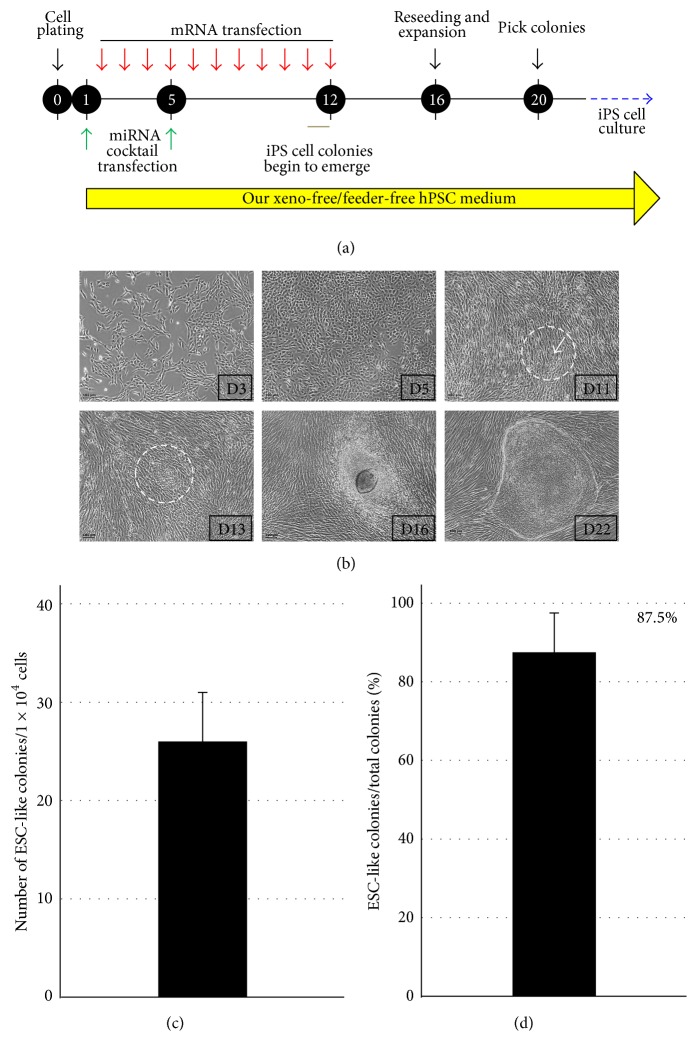
RNA-mediated generation of iPS cells using an ECM-based xeno-free/feeder-free hPSC medium. (a) A schematic representation of the experimental design. Human adult dermal fibroblasts were seeded on 6-well plates (5 × 10^4^ cells per well) and were subjected to multiple transfections as shown in the schedule. (b) The ESC-like colonies began to appear on day 11 after cell seeding (see the tip of the arrow at the center of the white circle,* top right panel*). The number within the square at the right bottom corner of each panel indicates the number of days passed after cell seeding. Scale bar: 100 *µ*m. (c) The graph shows the number of ESC-like colonies generated per 10,000 fibroblasts initially seeded. (d) The percentage of ESC-like colonies among the total colonies is presented in graph format.

In our experiment, we sought to generate xeno-free and integration-free iPS cells by combining our ECM (vitronectin) based xeno-free/feeder-free hPSC culture system with an mRNA-mediated reprogramming method. The mRNA reprogramming method used in this study was modified such that the reprogramming efficiency was enhanced using a microRNA cocktail. The microRNA cocktail was transfected into fibroblasts twice, on days 1 and 5 after cell seeding. A mixture of Oct4, Sox2, Klf4, c-Myc, and Lin28 mRNAs was transfected into the fibroblasts for 11 consecutive days beginning on day 2 after seeding. The phenomenon of the mesenchymal-to-epithelial transition (MET) was apparent between days 3 and 5 (Figure 1(b), top row, left two panels). The initial signs of ESC-like colonies (tightly packed cell clumps with clear borders and are composed of cells with high nucleus-to-cytoplasm ratio and notable nucleoli) began to appear on day 11 (Figure 1(b), top row, the most right panel), although they became more evident by day 13 (Figure 1(b), bottom row, the most left panel) after cell seeding. If the cells became too dense during days 14–16, they were split into 3 dishes. Each ESC-like colony was selected approximately on days 20–22 and was cultured using our vitronectin-based xeno-free/feeder-free hPSC culture system for expansion. The efficiency of ESC-like colony formation was approximately 0.2–0.3% (Figure 1(c)), and strikingly, the percentage of the ESC-like colonies among the total colonies was remarkably high (~87.5%) (Figure 1(d)). Among the ESC-like colonies that we selected, two clones, referred to as mRNA-iPSC2 and mRNA-iPSC11, were used for further experimentation. DNA fingerprint analysis confirmed that these iPS cells were derived from the original fibroblasts used for iPS cell generation rather than from contamination by other pluripotent stem cells (Supplementary Table  3).

Taken together, our results showed the high efficiency and accelerated kinetics of our reprogramming process.

### 3.2. iPS Cells Derived from Fibroblasts Displayed Typical Characteristics of Pluripotent Stem Cells

**Figure 2:**
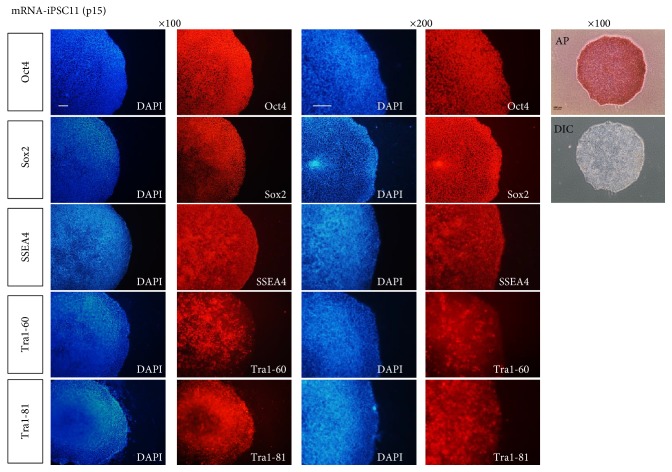
Immunostaining of the iPS cells for several pluripotent cell markers. The mRNA-iPSCs generated and cultured for 15 passages in our xeno-free/feeder-free hPSC system positively immunostained for the pluripotent cell markers Oct4, Sox2, SSEA4, Tra1-60, and Tra1-81. In addition, the colonies were stained with the commonly used undifferentiated cell marker alkaline phosphatase (*top right panel*).

The iPS cells that were passaged multiple times positively stained for alkaline phosphatase, an early marker of undifferentiated cells (Figure 2). All of the colonies exhibited sharp boundaries and rounded shapes, indicating their undifferentiated state. To more closely examine whether these iPS cells expressed markers of undifferentiated cells, immunostaining was performed for several antigens specific for PSCs. Oct4, Sox2, SSEA4, Tra-1-60, and Tra1-81 were robustly expressed in the iPS cells that had been passaged 15 times in our ECM-based xeno-free/feeder-free hPSC culture system (Figure 2). To examine the specificity of antibodies used in this study, we performed immunostaining of hESC-derived neural precursors with antibodies against some pluripotency markers such as Oct4 and Tra1-60 (Supplementary Figure  1).

**Figure 3:**
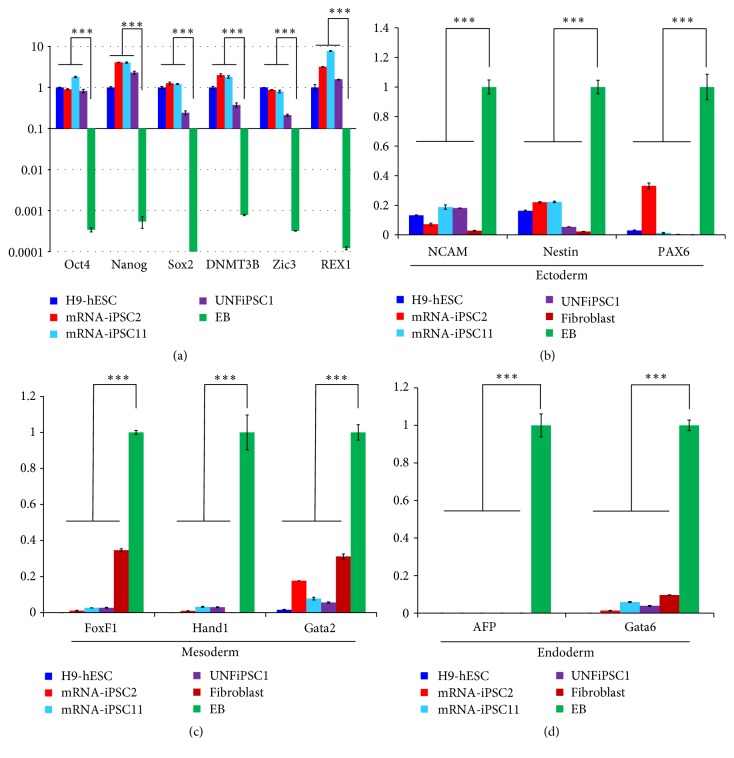
qRT-PCR analyses of the iPS cells. (a) The mRNA-iPSCs were analyzed for the expression of multiple representative lineage-specific markers. H9-hESCs and urine-derived iPS cells generated using the episomal plasmid method (UNFiPSC1) [18] were used as positive controls, and EBs were used as negative controls. ^*∗∗∗*^ *p* < 0.01. (b–d) The expression levels of representative markers of derivatives of ectoderm (NCAM, Nestin, and Pax6) (b), mesoderm (FoxF1, Hand1, and Gata2) (c), and endoderm (AFP and GATA6) (d) were examined via qRT-PCR. In these experiments, H9-hESCs and UNFiPSC1 were used as controls for undifferentiated cells, whereas EBs and fibroblasts were used as controls for differentiated cells. ^*∗∗∗*^ *p* < 0.01.

Next, we examined the expression levels of several markers of undifferentiated cells in two iPS cell lines generated in this study, along with hESCs and urine-derived iPS cells (UNFiPSC1) as positive controls. In addition, hESC-derived EBs were used as negative controls (Figure 3(a)). The mRNA-iPSCs expressed Oct4, Nanog, Sox2, DNMT3B, Zic3, and REX1 as abundantly as hESCs. In contrast, the mRNA-iPSCs minimally expressed representative markers for the ectoderm (NCAM, Nestin, and Pax6), mesoderm (FoxF1, Hand1, and Gata2), and endoderm (AFP and Gata6) lineages (Figures 3(b)–3(d)).

**Figure 4:**
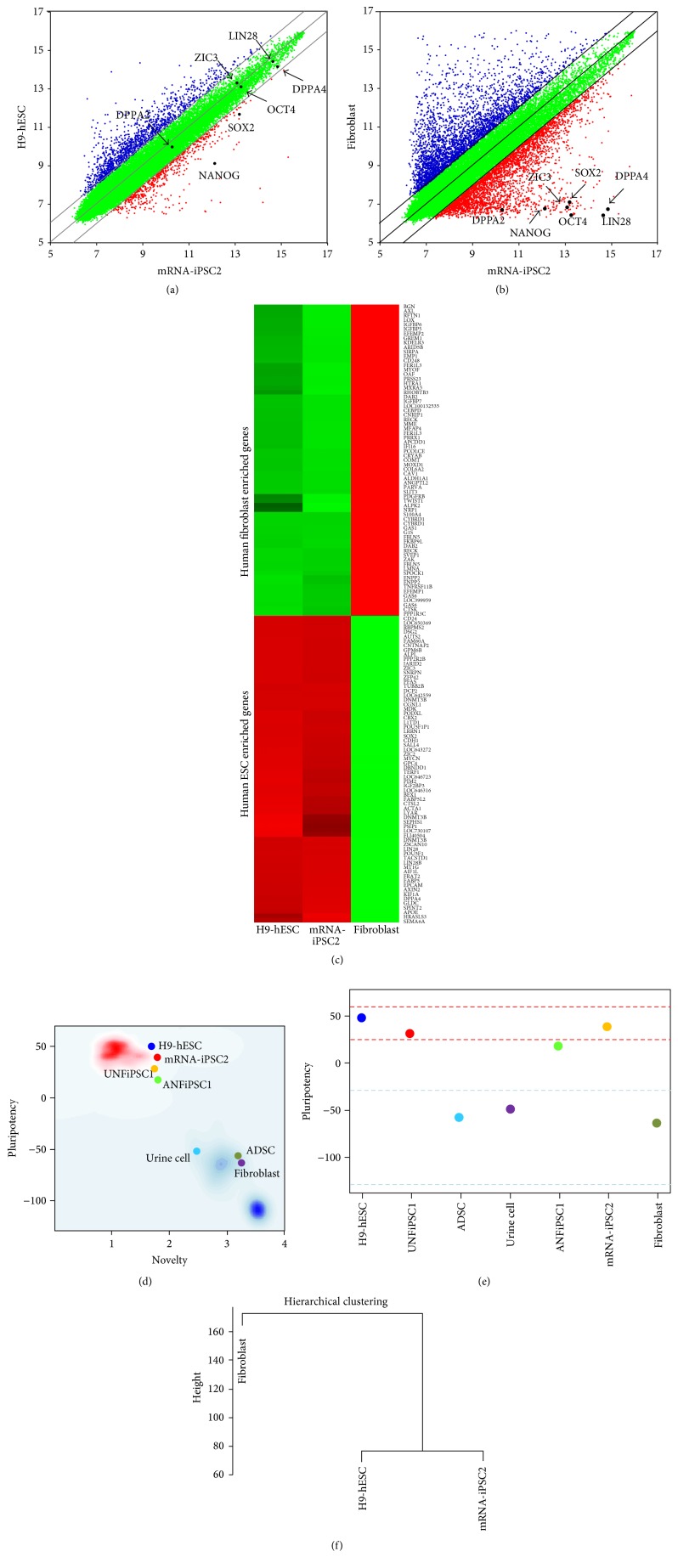
Genome-wide gene expression profiling of the mRNA-iPSCs. (a-b) Scatterplot analysis showed that the global gene expression pattern of mRNA-iPSCs is similar to that of H9-hESCs (a). However, the gene expression patterns are clearly different between mRNA-iPSCs and their parental cells, fibroblasts (b). (c) Heatmap analysis indicated that the expression patterns of 100 hESC-enriched genes and 100 human fibroblast-enriched genes (Supplementary Table  4) in mRNA-iPSCs were similar to those in H9-hESCs but not fibroblasts. (d-e) PluriTest analysis showed that mRNA-iPSCs and other previously established iPS cells (UNFiPSC1 and ANiPSC1) [18] were clustered with H9-hESCs in the pluripotent group, whereas fibroblasts and other primary cells were clustered in the nonpluripotent group. (f) Gene profiling-based hierarchical clustering analysis demonstrated the close association of the mRNA-iPSCs with H9-hESCs but not with the primary cells (fibroblasts).

We further analyzed the expression patterns of mRNA-iPSCs using a DNA microarray. Both scatterplot and heatmap analyses of the genome-wide gene expression profiles demonstrated that the gene expression pattern of the mRNA-iPSCs was very similar to that of hESCs (Figures 4(a) and 4(c)). However, the gene expression pattern of mRNA-iPSCs was significantly different from that of the fibroblasts from which the mRNA-iPSCs had originated (Figures 4(b) and 4(c)). The list of human ESC-enriched genes and fibroblast-enriched genes is presented in Supplementary Table  4.

PluriTest, a bioinformatic assay of pluripotency based on gene expression profiles, showed that the mRNA-iPSCs are pluripotent, similar to hESCs and other iPS cells, including UNFiPSC1 and ANFiPSC1 that have been established previously [18] (Figures 4(d) and 4(e)). Hierarchical clustering analysis of the gene expression data indicated that the mRNA-iPSC2 line was more similar to hESCs than to the fibroblasts from which this cell line was derived (Figure 4(f)).

### 3.3. Analysis of the Methylation Pattern of the Promoter Regions of Pluripotency-Associated Genes

**Figure 5:**
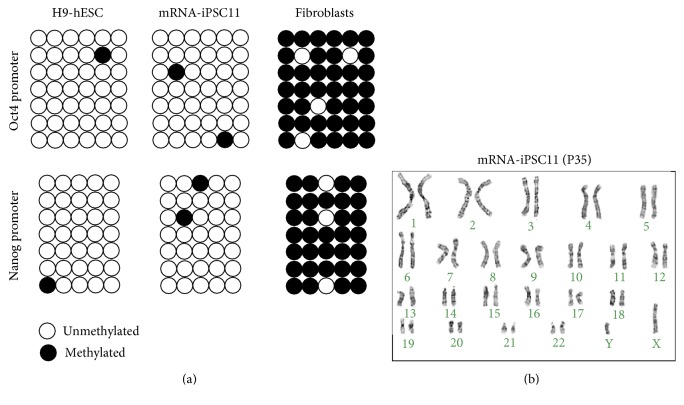
Analyses of the methylation in the Oct4 and Nanog promoters and of chromosomal abnormality in the mRNA-iPSCs. (a) The bisulfite sequencing data indicated that the promoters of the* Oct4* and* Nanog* genes in the mRNA-iPSCs were largely demethylated, similar to the methylation status of these promoters in hESCs. In contrast, their original cells, fibroblasts, were hypermethylated at these promoters. (b) G-banding analysis showed that no apparent chromosomal abnormality was generated during reprogramming and extended culture (for 35 passages) of the mRNA-iPSCs in our ECM-based xeno-free/feeder-free hPSC culture system.

We performed bisulfite sequencing analysis to examine the methylation patterns of the cytosine guanine dinucleotides (CpGs) in the promoters of two representative pluripotency-associated genes, Oct4 and Nanog. It is well documented that promoter methylation inversely correlates with gene expression [19]. The methylation of the promoter The DNA methylation patterns of the Oct4 and Nanog gene promoter regions of the mRNA-iPSCs were similar to those of the hESCs but not of the original fibroblasts (Figure 5(a)). This result is consistent with the high expression of the Oct4 and Nanog genes in both mRNA-iPSCs and hESCs (Figure 3(a)).

Cytogenetic analysis of Giemsa-banded metaphase mRNA-iPSCs showed no gross abnormality in the chromosomes, even after prolonged passaging up to passage 35 (Figure 5(b)).

### 3.4. Pluripotency of the mRNA-iPSCs Both* In Vitro* and* In Vivo*

**Figure 6:**
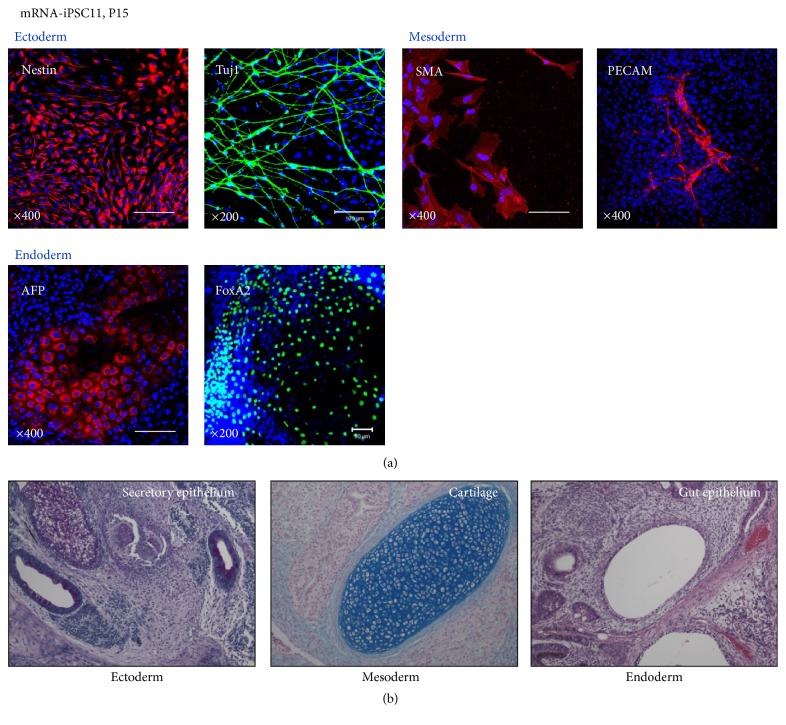
Analyses of pluripotency* in vitro* and* in vivo.* The mRNA-iPSCs were subjected to spontaneous differentiation via EB culturing, and the expression levels of representative markers of the ectoderm (Nestin and Class III *β*-tubulin (Tuj1)), mesoderm (SMA and PECAM), and endoderm (AFP and FoxA2) lineages were examined. Scale bar: 100 *µ*m. (b) Teratomas including structures and derivatives of the three germ layers were found at 3-4 months after intramuscular administration of the mRNA-iPSCs to NOD/SCID mice.

To examine whether the mRNA-iPSCs acquired pluripotency, the cells were spontaneously differentiated into the derivatives of the three germ layers* in vitro*. To this end, EBs were generated first, followed by the formation of adherent cultures on Matrigel-coated dishes in medium containing 10% FBS. When analyzed 15 days after differentiation on Matrigel, clear expression of representative markers for ectoderm (Nestin and Class III *β*-tubulin (Tuj1)), mesoderm (smooth muscle actin (SMA) and platelet endothelial cell adhesion molecule (PECAM)), and endoderm (alpha-fetoprotein (AFP) and FoxA2) was detected (Figure 6(a)).

When transplanted into NOD/SCID mice, the mRNA-iPSCs developed into teratomas consisting of various cell types derived from all three germ layers (secretory epithelium (ectoderm), cartilage (mesoderm), and gut (E4) epithelium (endoderm)) (Figure 6(b)).

Taken together, these results clearly demonstrate that the mRNA-iPSCs generated and cultured in our xeno-free and feeder-free hPSC culture system exhibited pluripotency.

## 4. Discussion

A human iPS cell-based clinical trial targeting retinal disease is currently underway [20], and several trials are expected to start within the foreseeable future. Therefore, the need for generating clinically compliant safe iPS cells continues to increase.

Among the methods for generating iPS cells without chromosome integration, mRNA transfection displays important advantages, such as the complete avoidance of chromosomal integration. Unlike the episomal transfection method, using the mRNA transfection method there is no need to examine the insertion of transgenes into the chromosome. In addition, mRNA transfection enables the rapid removal of exogenous genetic material from the cells after reprogramming, which reduces the unwanted negative effects of lingering transgenes.

However, this method is associated with certain problematic issues that must be resolved before its clinical application: (1) the original protocol for mRNA-mediated reprogramming required 17 daily transfections for complete reprogramming [11], which is highly labor-intensive; (2) the initial and subsequent protocols used either human feeder cells (i.e., human neonatal foreskin fibroblasts (NuFF)) [11] or NuFF-conditioned medium during the reprogramming stage [12, 15, 21, 22]. Using human feeder cells or NuFF-conditioned medium is both costly and laborious, requiring the repeated preparation of postmitotic human feeder cells and the collection of human feeder-conditioned medium, respectively; and (3) most of the mRNA reprogramming protocols reported to date have used different culture conditions for reprogramming and for expansion of the generated iPS cells. For example, Warren et al. used NuFF feeder cells during reprogramming and used mouse embryonic fibroblasts (MEFs) for expansion of the iPS cells [11]. More recent protocols for mRNA reprogramming adopted NuFF-conditioned medium during the reprogramming stage, followed by using either human feeder cells [21] or feeder-free defined medium [12] to culture the iPS cells. Using different culture systems for the reprogramming and expansion of iPS cells is inconvenient and cumbersome, prompting the search for a culture system that can be used throughout the entire process of iPS cell generation (i.e., both the reprogramming stage and the propagation of generated iPS cells).

In this study, we established a simpler and more convenient method for the generation and propagation of xeno-free and integration-free iPS cells by combining an mRNA transfection method with our previously developed xeno-free and feeder-free hPSC culture system. These two systems functioned compatibly during both the generation and expansion of xeno-free iPS cells. The mRNA transfection method used in this study was improved from the original protocol by adding two transfections of a microRNA cocktail during the reprogramming stage. These two transfections with the microRNA cocktail were considered to accelerate the kinetics and efficiency of reprogramming. Consistent with this concept, we detected ESC-like colonies beginning at 11 days after seeding the cells, which is faster than the results reported by Warren et al. (~day 17) [11]. The total number of daily mRNA transfections required for reprogramming was reduced from 17 [11] to 11 due to the transfection of the microRNA cocktail. Furthermore, we found that the percentage of ESC-like colonies among the total colonies was approximately 87.5%, which was significantly higher than previously reported results [2, 9]. Taken together, our results showed that a mixture of microRNAs facilitated the reprogramming process and, thus, rendered the mRNA-mediated generation of iPS cells as more labor- and time-effective.

Another important feature of our method is the use of the same (or very similar) xeno-free/feeder-free hPSC culture medium for both reprogramming and propagation of iPS cells, which makes the iPS cell generation and expansion procedures simpler and more convenient. The simplicity of our method will advance the production of good manufacturing practice (GMP) grade xeno-free iPS cells for cell therapy.

The efficiency of iPS cell generation using an mRNA reprogramming method has been reported to be significantly variable (0.04–4.4%) [11, 12, 22], which may be at least partially caused by the innate properties of the somatic cells used for reprogramming, the transfection efficiency, the number of reprogramming factors introduced, the oxygen concentration during culturing (5% vs. 20%), and the culture media/conditions adopted for reprogramming [11, 12, 22]. For example, the reprogramming efficiency, which was 1.89% on feeder cells, dropped to 0.22% when a Matrigel-based feeder-free culture condition was used [22]. In our study, we consistently obtained a reprogramming efficiency of ~0.2–0.3% under normoxic conditions (20% O_2_) when our xeno-free/feeder-free hPSC culture system was adopted for the reprogramming of human adult dermal fibroblasts.

The medium used in this study was a completely defined, xeno-free, and feeder-free hPSC culture medium. Here, we demonstrated that this medium was highly compatible with a modified mRNA transfection method for the generation and expansion of iPS cells. Therefore, this combination of an ECM-based xeno-free/feeder-free hPSC culture system with an improved version of the mRNA transfection method provides a rapid, efficient, and labor-effective platform for the generation of footprint-free and xeno-free iPS cells for safe clinical applications.

## 5. Conclusions

Here, we report that therapeutically safe (i.e., xeno-free and footprint-free) iPS cells can be efficiently generated and expanded by combining our previously developed ECM-based xeno-free/feeder-free hPSC culture system with an improved mRNA reprogramming method, facilitating the use of a labor- and time-effective platform for future iPS cell-mediated cell therapy.

## Supplementary Material

Supplementary Table 1: List of primers used in this study. Supplementary Table 2. List and description of the antibodies used for immunostaining. Supplementary Table 3. DNA fingerprinting analysis Supplementary Table 4. List of the hESC-enriched genes and fibroblast-enriched genes shown in Figure 4(C). Supplementary figure 1. Immunostaining of hESC-derived neural precursors for pluripotency markers, OCT4 and TRA1-60.
