# Supplementary material for: Extracellular Matrix-Dependent Generation of Integration- and Xeno-Free iPS Cells Using a Modified mRNA Transfection Method
Source: Stem Cells Int. 2016 Jan 12;2016:6853081. doi: 10.1155/2016/6853081 (PMC4737460; doi:10.1155/2016/6853081)
Supplement: Supplementary file 1 — Supplementary Table 1: List of primers used in this study. Supplementary Table 2. List and description of the antibodies used for immunostaining. Supplementary Table 3. DNA fingerprinting analysis Supplementary Table 4. List of the hESC-enriched genes and fibroblast-enriched genes shown in Figure 4(C). Supplementary figure 1. Immunostaining of hESC-derived neural precursors for pluripotency markers, OCT4 and TRA1-60. [file 6853081.f1.zip › supplementary figure 1.pptx]

## Slide 1
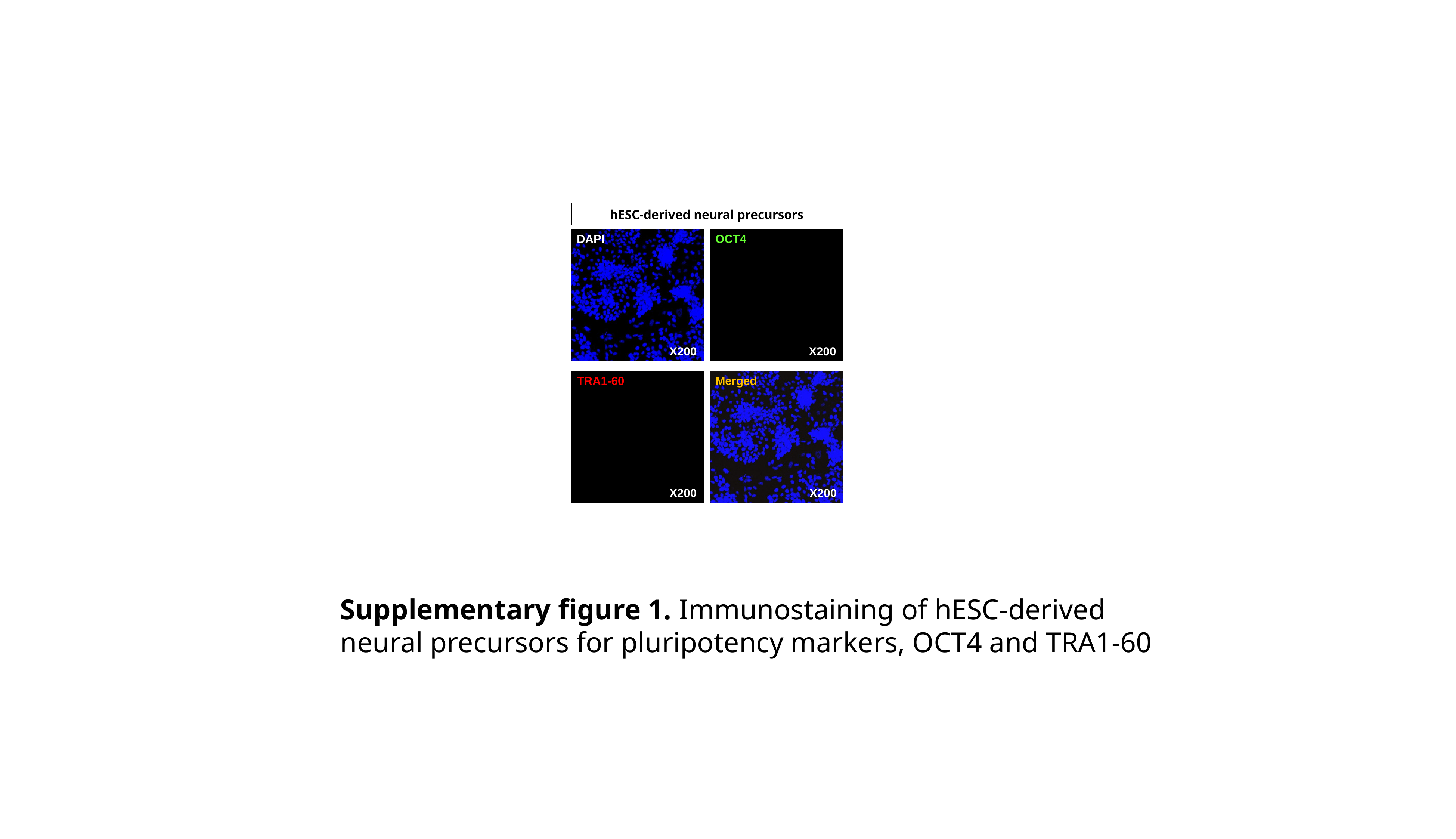

hESC-derived neural precursors
DAPI
OCT4
X200
X200
Merged
TRA1-60
X200
X200
Supplementary figure 1. Immunostaining of hESC-derived
neural precursors for pluripotency markers, OCT4 and TRA1-60
